# Case Report: Successful treatment of severe type II decompression sickness characterized by multiple gas emboli

**DOI:** 10.3389/fmed.2025.1690176

**Published:** 2025-12-05

**Authors:** Yan Wang, Yujie Wang, Shuping Li, Donghua Ai, Yubo Chen, Mei Jing

**Affiliations:** 1 Naval Medical Center, Naval Medical University, Shanghai, China; 2 The Second Hospital of the Navy of the Southern Theater Command, Sanya, China

**Keywords:** diving operation, type II decompression sickness, multiple gas emboli, decompression protocols, recompression therapy

## Abstract

**Background:**

Decompression sickness (DCS) is usually caused by inadequate decompression. Although adherence to decompression protocols can significantly reduce the incidence of DCS, it still cannot prevent all cases from occurring. If a large number of gas bubbles enter the right heart and pulmonary arterial system, patients may present with symptoms such as cough, tachypnea, chest pain, dyspnea, or even shock. The presence of numerous bubbles in the abdominal cavity and portal venous system may also lead to liver dysfunction or abdominal pain. Theoretically, DCS occurring after dives that follow decompression tables should be relatively mild. The development of severe Type II DCS characterized by multiple gas emboli following protocol adherence is considered rare.

**Case presentation:**

We report a case of a diver who developed severe Type II DCS characterized by multiple gas emboli despite conservative adherence to a decompression protocol. The maximum dive depth was 19 meters, with a total dive duration of 120 min. His underwater task involved heavy lifting, and he performed decompression conservatively according to the Chinese Air Diving Decompression Table for decompression, with a total decompression time of 45 min. However, 30 min after surfacing, the patient developed symptoms including chest tightness, shortness of breath, dyspnea, fatigue, and pain in the left knee and thigh. Computed tomography (CT) scans of the chest and abdomen revealed gas emboli in multiple locations, including the pulmonary artery, right ventricle, and hepatic portal vein. The patient recovered completely after timely recompression therapy and was discharged.

**Conclusion:**

This report highlights the unpredictability of DCS; even when decompression tables are followed, severe Type II DCS may occur if the diver’s underwater workload is excessive and multiple risk factors are present. Prompt recompression therapy is crucial to prevent clinical deterioration. Due to the limitations of current DCS models, further research is needed to develop individualized safe decompression protocols based on physiological variables.

## Introduction

Decompression sickness (DCS) is a disease caused primarily by the formation of bubbles from dissolved gas in the blood and/or tissues following a reduction in ambient pressure (1, 2). It can cause symptoms such as joint pain, paresthesia, cutaneous manifestations, and cardiopulmonary dysfunction (3, 4). According to naval medical standards, DCS is classified as mild Type I (Pain, Lymphatic or Skin, Constitutional or Nonspecific) and serious Type II (serious neurological, cardiopulmonary, mild neurological) (5). DCS is usually triggered by inadequate decompression. Although strict adherence to decompression protocols can significantly reduce the incidence of DCS, it cannot completely prevent all cases. If a large number of bubbles enter the right heart and pulmonary arterial system, patients may experience coughing, shortness of breath, chest pain, difficulty breathing, or even shock (6). DCS affecting the cardiopulmonary system is a severe form of Type II DCS. Such severe manifestations often result from errors in diving operations, such as skipping required decompression stops or ascending too rapidly (7), making it a critical emergency that requires immediate recompression therapy. The presence of numerous bubbles in the abdominal cavity and portal venous system may also cause impaired liver function or abdominal pain. Theoretically, cases of DCS occurring despite compliance with decompression protocols tend to be relatively mild. The occurrence of severe Type II DCS characterized by multiple gas emboli after conservative decompression is uncommon.

This report describes a diver who developed severe Type II DCS with multiple gas emboli despite conservative adherence to a decompression protocol. We detail the dive history, medical history, symptoms, signs, and results of various examinations. The patient was diagnosed with Type II DCS and successfully discharged after effective recompression and adjunctive therapy.

## Case presentation

**Figure 1:**
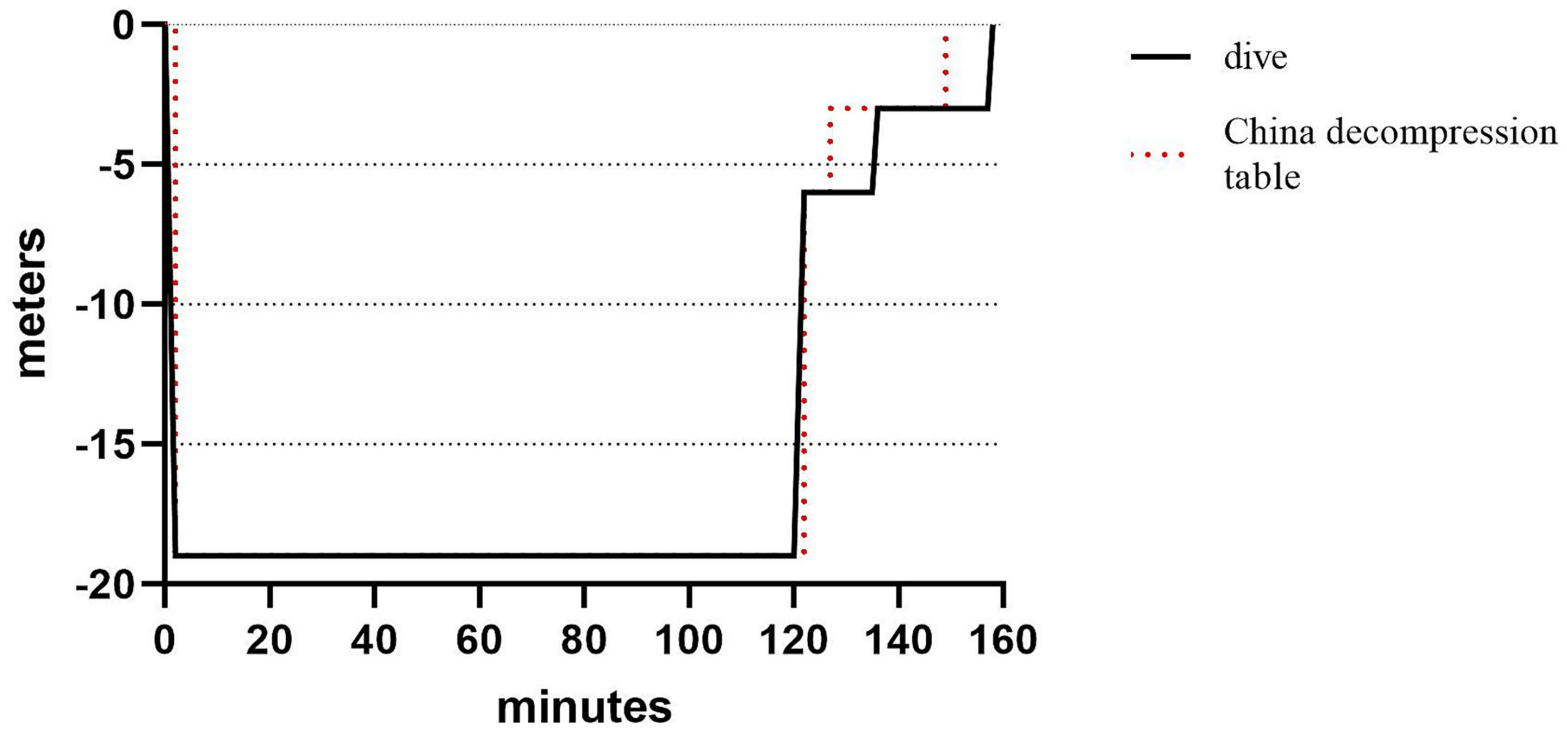
Comparison of the patient’s decompression procedure with the Chinese Air Diving Decompression Table. The horizontal axis represents “total underwater duration (minutes),” and the vertical axis represents “dive depth (meters).” The solid line indicates the standard decompression time of the Chinese Air Diving Decompression Table, and the dashed line indicates the patient’s actual decompression time (45 min, 16 min longer than the standard).

On June 2, 2025, a 51-year-old Chinese male commercial diver performed diving operations in the northern South China Sea. The diver used a surface-supplied air system, with compressed air as the breathing gas, reaching a maximum depth of 19 meters and spending a total of 120 min underwater. His main task involved moving heavy objects underwater. After completing the work, the diver underwent in-water staged decompression, which included two stops: the first at 6 meters for 8 min, and the second at 3 m for 26 min. The entire decompression process lasted 45 min (Figure 1). Due to the heavy workload underwater that day, and to better eliminate nitrogen, he extended the decompression stop time based on his experience. The total decompression time exceeded the China decompression table for air diving by 16 min. Thirty min after surfacing, the patient developed chest tightness, dyspnea, fatigue, and pain in the left knee and thigh. No petechiae were observed on the skin, and there was no loss of consciousness, headache, nausea, or vomiting.

The patient was transported to our hospital by ambulance. During transit, he received high-concentration oxygen, which alleviated his dyspnea, but chest tightness, fatigue, and limb pain persisted. Upon admission, a diving physician conducted a detailed history and physical examination. The patient had been a commercial diver for 20 years with no history of chronic diseases or previous diving-related illnesses. On the night before the dive, he consumed over 100 g of white liquor. The underwater work intensity was significantly higher than usual due to poor water conditions and strong currents.

Physical Examination on Admission: Temperature 36.5°C, blood pressure 115/79 mmHg, pulse 76 beats/min, respiratory rate 19 breaths/min. Height 178 cm, weight 90.3 kg, BMI 28.3. No abnormal petechiae or ecchymoses were observed on the skin. Cardiopulmonary and abdominal examinations were unremarkable (despite the complaint of chest tightness). Neurological examination revealed no positive signs. Musculoskeletal examination showed tenderness in the left knee and thigh without swelling or erythema.

**Figure 2:**
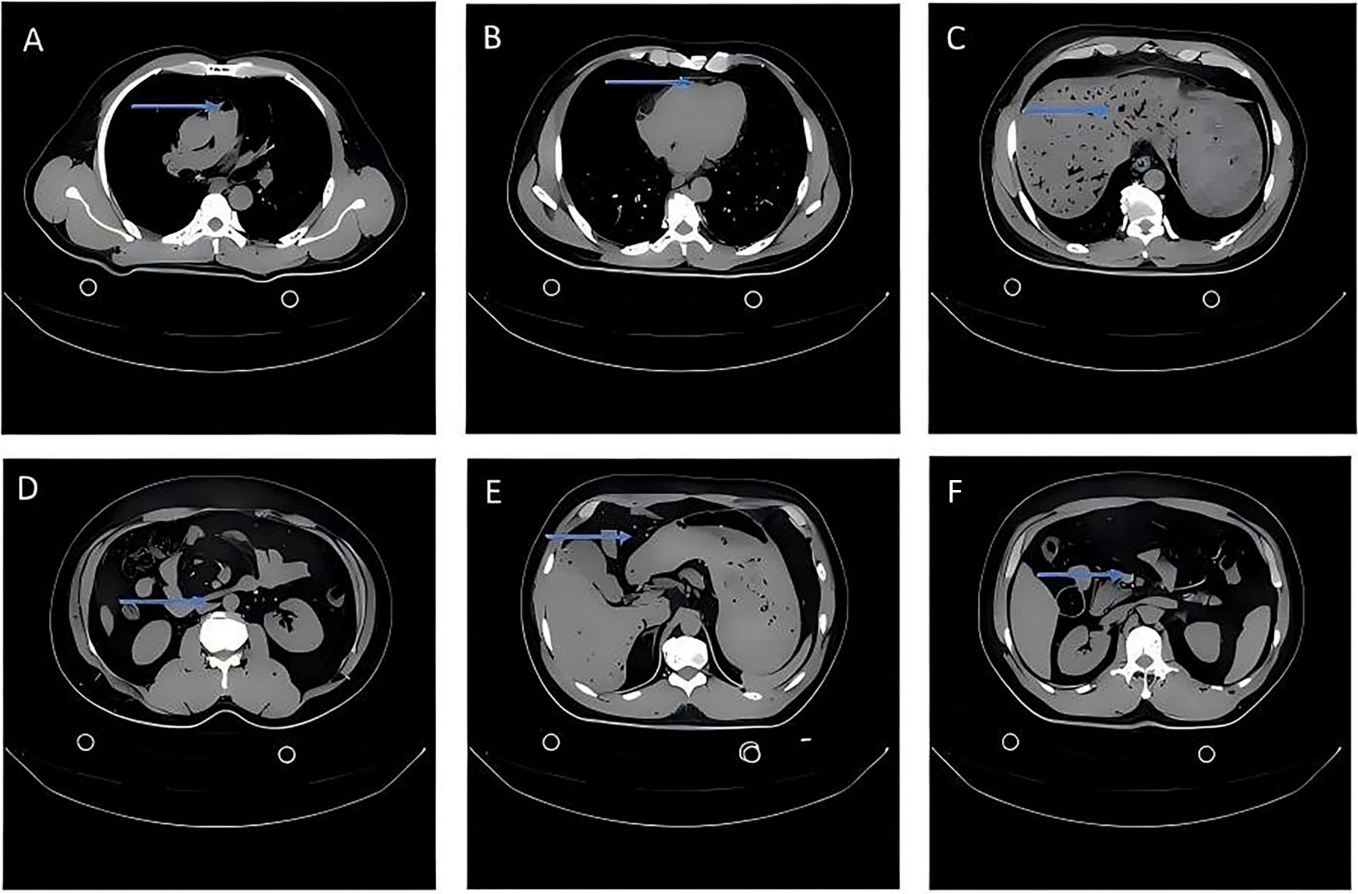
Axial CT images demonstrating air emboli. **(A)** Gas bubble in the pulmonary artery trunk (arrow). **(B)** Bubble in the right atrium (arrow). **(C)** Extensive gas within the intrahepatic portal venous system (arrows). **(D)** Gas bubble in the inferior vena cava (arrow). **(E)** Intraperitoneal free gas (arrow). **(F)** Gas bubble in the superior mesenteric vein (arrow). CT images of chest and abdominal gas emboli (non-contrast, mediastinal window). **(A)** Gas bubbles in the pulmonary artery trunk (arrow, associated with dyspnea). **(B)** Gas bubbles in the right atrium (arrow). **(C)** Extensive gas in the hepatic portal venous system (arrow, potential cause of liver function impairment). **(D)** Gas bubbles in the inferior vena cava (arrow). **(E)** Pneumoperitoneum (arrow, may cause abdominal pain). **(F)** Gas bubbles in the superior mesenteric vein (arrow).

Emergency blood tests, arterial blood gas analysis, and immediate CT scans of the head, chest, and upper abdomen were performed. Results of arterial blood gasses and routine blood tests are shown in Table 1. CT scans revealed multiple gas emboli in the portal vein branches, superior mesenteric vein, inferior vena cava, pulmonary artery, and right ventricle, along with several intra-abdominal gas shadows (Figure 2). Brain CT showed no significant abnormalities. Based on the clinical presentation, decompression process, time of symptom onset (30 min after surfacing), and imaging findings, the patient was diagnosed with Type II DCS. Alternative diagnoses for chest tightness and fatigue, including acute coronary syndrome, pulmonary embolism, and pneumothorax, were ruled out by normal ECG, negative cardiac enzymes, and absence of corresponding findings on CT imaging.

**Table 1:** Key laboratory findings on admission.

Test item	Result	Reference range	Notes
Arterial blood gas			
pH	7.36	7.35–7.45	
PCO₂, mmHg (Kpa)	41.4(5.52)	35–48	
PO₂, mmHg (Kpa)	128.3(17.11)	80–100	
O₂ Sat, %	98.8	>95	
Lactate, mmol/L	3.4	0.5–1.0	Elevated
Complete blood count			
WBC, ×10⁹/L	10.04	4.0–10.0	Elevated
Neutrophil %	75.1	50–70	Elevated
Hemoglobin, g/dL	16.8	12–16	Elevated
Platelets, ×10⁹/L	113	125–350	Decreased
Hematocrit, %	51.2	40–50	Elevated
Serum biochemistry			
hs-CRP, mg/L	2.83	0–4	
ALT, IU/L	53	<40	Elevated
AST, IU/L	56	<40	Elevated
GGT, IU/L	136	8–61	Elevated
CK, IU/L	192	50–310	
Creatinine, μmol/L	78	59–104	
Random glucose, mmol/L	12.57	<7.8	Elevated
Lipid profile			
Triglycerides, mmol/L	2.15	<1.70	Elevated
Total cholesterol, mmol/L	6.82	<5.20	Elevated
LDL, mmol/L	4.21	<3.40	Elevated

Technique: Axial images were obtained from the thoracic inlet to iliac crests without intravenous contrast (SOMATOM Sensation 16, Siemens, Germany; scan with 98 effective mAs, 140 kVp, and 5.0 mm slice thickness).

**Figure 3:**
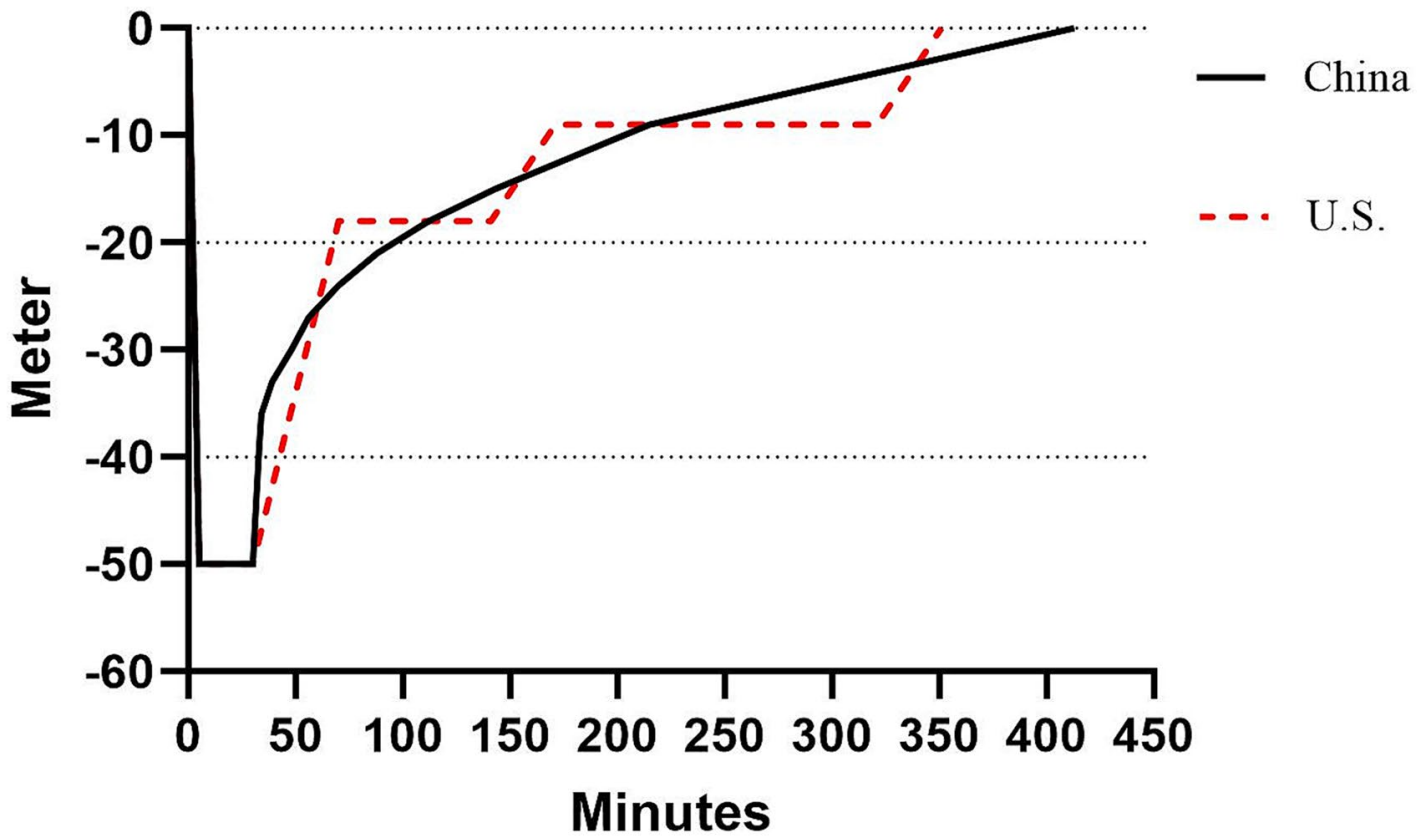
Comparison between the Chinese Naval Medical University Air Treatment Table IV and the U. S. Navy Treatment Table 6A for DCS. The horizontal axis represents “treatment duration (minutes),” and the vertical axis represents “treatment pressure (Meters of seawater, corresponding to ATA).” The two curves differ in pressure reduction rate and total treatment time, with the Chinese protocol showing a slower pressure reduction rate in the later stage.

Given that the patient had clinical manifestations such as left knee and thigh pain but no signs of neurological impairment, he was concurrently diagnosed with acute type I DCS alongside acute type II DCS. Conventional treatment was initiated, including continuous oxygen therapy and fluid resuscitation (1,000 mL of 0.9% sodium chloride solution intravenously) (8). Steroids were not administered. Two hours after symptom onset, recompression therapy began using the Chinese Naval Medical University Air Treatment Table IV. Symptoms including chest tightness, shortness of breath, fatigue, and limb pain significantly improved when pressure reached 5 ATA. The total treatment duration according to the Chinese Diving Decompression Table 5 protocol was approximately 6 h (air above 18 m; intermittent pure oxygen below 18 m). Differences between this treatment table and the U. S. Navy Treatment Table 6A are illustrated in Figure 3. Symptoms resolved completely after recompression.

Subsequent tests revealed elevated blood pressure and blood glucose. Fasting glucose levels fluctuated between 7 and 13 mmol/L, with an HbA1c of 7.2%. Blood pressure ranged from 140 to 160/90–99 mmHg. Additional diagnoses included: Obesity (by modified Asian criteria), Type 2 Diabetes Mellitus, Hypertension, and Hyperlipidemia. A transthoracic echocardiogram (TTE) with bubble study performed after admission did not detect a patent foramen ovale (PFO). On the second and third hospital days, to consolidate therapeutic efficacy, conventional hyperbaric oxygen therapy (HBOT) was administered following the 2018 guidelines of the Chinese Hyperbaric Oxygen Medicine Society. This involved sessions at 2.0–2.5 ATA, once daily for 60–90 min each, using a multi-place chamber compliant with Chinese National Standard GB/T 12130. After two HBOT sessions, a repeat abdominal CT confirmed complete resolution of the gas emboli. Laboratory parameters normalized, the patient was asymptomatic, and he was discharged cured. One week after discharge, a follow-up phone call confirmed a good recovery with no sequelae.

## Discussion

This patient followed a decompression table with stops and ascended slowly without recorded breath-holding or rapid ascent. Furthermore, chest CT showed no radiological evidence of pulmonary barotrauma but revealed bubbles in the right ventricle, pulmonary artery, portal vein, and other locations. Therefore, the diagnosis was acute Type II DCS rather than arterial gas embolism (AGE), which typically presents with immediate and dramatic symptoms, with symptom latency exceeding 10 min being exceptionally rare (9). Bubble formation is considered the primary injurious mechanism in DCS. In this case, the patient’s chest tightness and dyspnea, coupled with CT findings of bubbles in the pulmonary artery and right ventricle, suggest transient cardiopulmonary dysfunction related to the gas emboli. If untreated or with a larger bubble load, pulmonary artery pressure can increase in a dose-dependent manner, potentially leading to acute right heart failure (10). Such severe presentations usually follow significant decompression violations or rapid ascents. Although the patient underwent a diving procedure with conservative decompression, the exceptionally long bottom time and high exertion level led to a high inert gas load and significant decompression stress. Reports of severe Type II DCS with multiple emboli following such compliant dives remain rare.

Admission laboratory tests showed hyperlactatemia on arterial blood gas analysis, but other parameters were normal. Lactate is produced under normal physiological conditions and often elevated in various pathological states, indicating impaired tissue oxygenation due to reduced oxygen delivery or mitochondrial dysfunction. Significantly elevated lactate can have serious hemodynamic consequences and may serve as a potential marker of severity. The normal pH, PaO_2_, and PaCO_2_ were likely because the number of intravascular bubbles was insufficient to cause major flow obstruction and severe cardiopulmonary dysfunction. Mild leukocytosis, elevated hematocrit, and mild thrombocytopenia are consistent with the clinical picture of DCS. Studies indicate that bubbles can activate platelets (11, 12), complement (13), and the coagulation system (14). Leukocytes may aggregate at bubble surfaces and be activated by bubble-induced endothelial damage (15). Another important activation mechanism associated with DCS is an increase in circulating pro-inflammatory microparticles (16). It is speculated that inflammatory activation might contribute to atypical DCS symptoms like fatigue or malaise. Furthermore, inflammatory mechanisms likely explain the hemoconcentration and shock occasionally seen in fulminant DCS. All these laboratory parameters normalized before discharge. Diagnoses of hypertension, dyslipidemia, and diabetes were based on international medical standards. CT scans revealed gas accumulation in the portal vein and branches, superior mesenteric vein, inferior vena cava, pulmonary artery, and pericardial area around the right ventricle, alongside several intra-abdominal gas shadows. All abnormalities resolved completely after treatment. Bubbles forming in the portal system first reach the liver; significant quantities can cause gas trapping in the portal venous system, potentially accompanied by abdominal pain and sometimes liver injury.

Based on this case, we propose the following potential mechanisms explaining the severe Type II DCS with multiple emboli despite conservative decompression: 1. Individual Susceptibility: Significant inter- and intra-individual variability exists in bubble formation and decompression symptoms. Analysis of 320 DCS cases from a specific DAN database segment identified factors including female sex, older age, high BMI, high body fat, greater depth, high gradient factors, high water temperature, strenuous pre-dive activity, high underwater workload, and use of a drysuit (17). This patient’s advanced age, high BMI, and high workload on the dive day were likely major contributing factors. Notably, consuming 100 g of alcohol the night before the dive likely caused dehydration, impairing inert gas elimination (18, 19). Furthermore, vascular integrity and optimal function are crucial for gas exchange during diving (20). This patient’s comorbid hypertension and Type 2 diabetes suggest impaired endothelial function, potentially increasing DCS risk. While a patent foramen ovale (PFO) is a common risk factor for DCS (21), bubble contrast TTE in this case was negative. Although TTE is less sensitive than transesophageal echocardiography (TEE) for bubble detection, its non-invasive nature makes it the most common screening tool for right-to-left shunts (RLS) (22). While a PFO cannot be entirely ruled out, its relevance to cardiopulmonary DCS may be limited in this instance. 2. Limitations of DCS models: Decompression tables are primarily developed using two methods: probabilistic (analyzing historical dive data to select acceptably safe depth-time combinations) and deterministic (using physiological gas models to estimate DCS risk) (23). Tables and dive computers use the concept of “leading tissues” to calculate decompression stop depth and duration, aiming to control DCS risk (24). However, even with empirical or computational decompression protocols, DCS cannot be completely prevented. Such cases are often deemed unpredictable, highlighting persistent limitations in the predictive power of current algorithms.

Consequently, diving medicine experts increasingly suspect that bubble formation and DCS manifestation may relate not only to the dive itself but also to pre-dive conditions (25, 26) and individual susceptibility, a link observed in other diving-related disorders (27). The relationship between bubbles and DCS is more complex than previously thought; other variables like endothelial function (25, 26), hydration status (19), and vascular/lymphatic responses may be involved in severe cases (28, 29). We believe increased research efforts are needed to elucidate the complex pathophysiology of decompression. The reliability limits of current dive computer validation protocols may have been reached. A discrepancy exists between physiological decompression requirements and the schedules generated by tables/computers. Enhancing the ability to tailor safe decompression based on physiological variables is crucial. This could be based on pre-determined data from existing scientific evidence, like the factors mentioned above, or, in the foreseeable future, through real-time physiological sensor-assisted diver-computer interaction.

This report emphasizes the unpredictability of DCS. Severe Type II DCS can occur even with table adherence, particularly with prolonged underwater exertion and multiple patient risk factors. Accurate diagnosis of atypical symptoms in divers is crucial for critical care and emergency physicians. Prompt recompression therapy can halt rapid progression, and delayed treatment can still be effective. Further research is needed to develop individualized decompression strategies based on physiological variables.

## Data Availability

The datasets presented in this study can be found in online repositories. The names of the repository/repositories and accession number(s) can be found in the article/supplementary material.
